# Phase Shift Compensation Enables Rainbow‐Free, Ultrawide Angular, and Pattern‐Free Nonreciprocal Thermal Emission

**DOI:** 10.1002/nap2.70075

**Published:** 2026-04-15

**Authors:** Kaixia Xu, Kezhang Shi, Meiya Rong, Run Hu, Chengping Yin

**Affiliations:** ^1^ Key Laboratory of Atomic and Subatomic Structure and Quantum Control (Ministry of Education) School of Physics Guangdong Provincial Key Laboratory of Quantum Engineering and Quantum Materials Guangdong‐Hong Kong Joint Laboratory of Quantum Matter Frontier Research Institute for Physics South China Normal University Guangzhou China; ^2^ Ningbo Global Innovation Center Zhejiang University Ningbo China; ^3^ Centre for Optical and Electromagnetic Research National Engineering Research Center for Optical Instruments Zhejiang University Hangzhou China; ^4^ School of Information Science and Engineering NingboTech University Ningbo China; ^5^ School of Energy and Power Engineering Huazhong University of Science and Technology Wuhan China

**Keywords:** nonreciprocal Fabry‐Perot resonance, pattern‐free nonreciprocal thermal emission, phase shift compensation, rainbow‐free, ultra‐wide angular

## Abstract

Thermal emission from a conventional blackbody is inherently incoherent. Non‐Kirchhoff thermal emitters, which exploit nontrivial nonreciprocal properties, enable unprecedented control over both the spatial and temporal coherence of thermal emission with enhanced degrees of freedom. Although violations of Kirchhoff's law have been demonstrated, achieving strong nonreciprocity while suppressing the rainbow effect across ultrawide angular ranges remains an open challenge. This study demonstrates a pattern‐free Fabry–Pérot resonator comprising Weyl semimetal/germanium heterostructures on a silver substrate that achieves rainbow‐free nonreciprocal thermal emission with exceptional angular stability. The Weyl semimetal serves dual functions: simultaneously breaking Lorentz reciprocity and providing dynamic phase shift compensation, enabling the structure to maintain nonreciprocity exceeding 0.6 or 0.8 across an ultrawide angular range of Δ*θ* = 75° or 60° at a wavelength of 11.17 μm, substantially surpassing current state‐of‐the‐art narrowband nonreciprocal emitters in both angular coverage and monochromaticity (Δ*λ*/*λ*_0_ < 0.54%). Active tuning of the resonant wavelength is achieved through either chemical potential modulation in the Weyl semimetal or precise cavity thickness control, without compromising the rainbow‐free or ultrawide angular response. Characterization of thermal and azimuthal dependencies reveals exceptional stability across wide temperature (Δ*T* = 100 K) and azimuthal angular ranges (Δ*δ* = 60°). These findings enable simplified, low‐cost thermal emitters with potential applications in infrared imaging, sensing, and energy harvesting.

## Graphical abstract

A pattern‐free nonreciprocal emitter is demonstrated, exhibiting strong nonreciprocity (*η* > 0.6 or 0.8) across an unprecedented angular range (Δ*θ* = 75° or 60°) at the target wavelength with monochromaticity of Δ*λ*/*λ*₀ < 0.54%. The wavelength is tunable without compromising the rainbow‐free or ultrawide angular response, establishing a generalizable design framework for achieving target performance at other wavelengths.

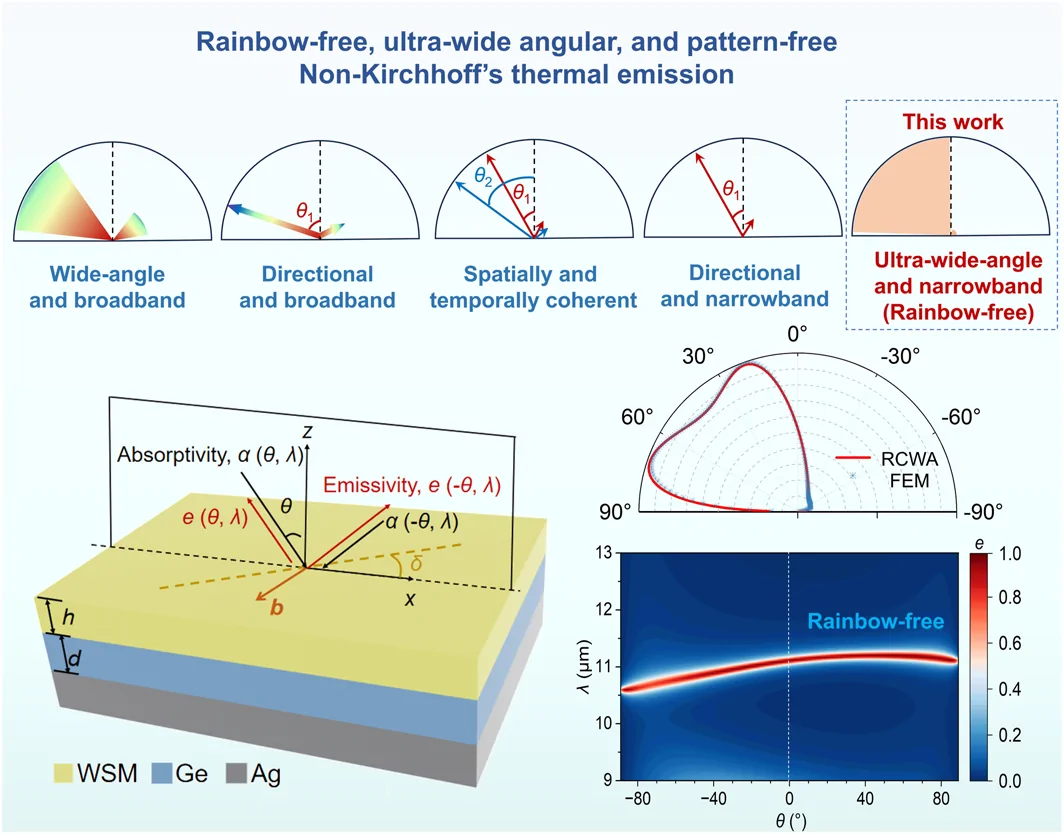

## 1 Introduction

Thermal emission serves as the principal mechanism of energy exchange between macroscopic objects [1, 2], forming the physical basis for essential technologies including solid‐state lighting [3, 4], radiative cooling systems [5, 6], and thermal camouflage [7, 8]. Kirchhoff's law of thermal emission, which establishes the equivalence between spectral directional emissivity and absorptivity for a given polarization state, has been the cornerstone for the design and analysis of thermal emitters. This fundamental principle allows the determination of emissivity through absorption measurements, thereby avoiding the need for a complex calculation based on fluctuational electrodynamics [9]. Nevertheless, such reciprocity imposes fundamental limitations on thermal emission control and fails to describe nonreciprocal systems [10, 11], creating both theoretical constraints and significant engineering challenges. This limitation becomes particularly evident in conventional solar absorbers, where high absorption efficiency necessarily leads to significant thermal emission losses, fundamentally restricting the maximum achievable energy collection efficiency below the Landsberg limit [12, 13, 14]. Such an inherent trade‐off underscores the critical need for breaking the emissivity–absorptivity equivalence to overcome fundamental thermodynamic constraints in energy conversion systems.

Recent advances have established multiple pathways to overcome Kirchhoff's law constraints, primarily through (i) breaking of time‐reversal symmetry [15], (ii) harnessing of nonlinear optical effects [10], and (iii) integration of nonreciprocal materials, such as magnetized InAs [16], magnetized InSb [17], and magnetic Weyl semimetals (WSMs) [17]. These fundamental approaches have yielded broadband nonreciprocal thermal emission with directional or wide‐angle characteristics [16, 17, 18] (Figure 1aI,II) and spectrally selective narrowband emitters with tailored thermal properties [19, 20, 21, 33]. Recently, although previous studies have explored alternative mechanisms for achieving narrowband and wide‐angle nonreciprocal thermal emission, achieving rainbow‐free, ultrawide‐angle, and pattern‐free nonreciprocal thermal emission through a simpler yet more efficient approach remains an open challenge. For example, Wang et al. demonstrated narrowband, wide‐angle nonreciprocity using a periodic InAs grating embedded within a metallic cavity [22], an approach that differs fundamentally from our scheme and presents notable fabrication challenges. Separately, an omnidirectional nonreciprocal emitter has been proposed using a complex multilayer stack of ten layers [23]. However, the existing narrowband nonreciprocal emitters are constrained by their limited angular response (typically < 50°) and reliance on complex nanostructured designs, as evidenced in recent studies [19, 22, 23, 24, 25] (Figure 1aIII,IV). These limitations highlight the pressing need for alternative approaches that can simultaneously achieve rainbow‐free emission [34, 35] and wide‐angle nonreciprocal performance while eliminating the requirement for intricate subwavelength patterning and highly multilayered stacking architectures [23]. The development of simplified yet high‐performance thermal emitters remains an ongoing challenge, which is also fundamentally crucial for enhancing the energy conversion efficiency of thermophotovoltaic systems with selective wavelengths. Specifically, the narrowband characteristic enables spectral selectivity within the key operational band of photovoltaic cells, thereby minimizing energy losses outside the target wavelength range. Meanwhile, the wide‐angle nonreciprocal performance, which is characterized by high emissivity, for example, in directions from 0° to 89° and low emissivity from −89° to 0°, ensures significant angular asymmetry under realistic solar irradiation, particularly in concentrator systems where light is incident from varying angles. This angular asymmetry helps mitigate stray radiative losses from multiple directions and sustains effective directional control of thermal emission. The synergistic combination of spectral and spatial nonreciprocity represents a pivotal design paradigm for advancing solar absorbers beyond conventional efficiency limits [13, 36, 37]. Beyond its application in solar energy harvesting, the proposed rainbow‐free, wide‐angle nonreciprocal thermal emitter holds broader relevance in domains such as thermal signal detection and adaptive thermal camouflage.

**FIGURE 1:**
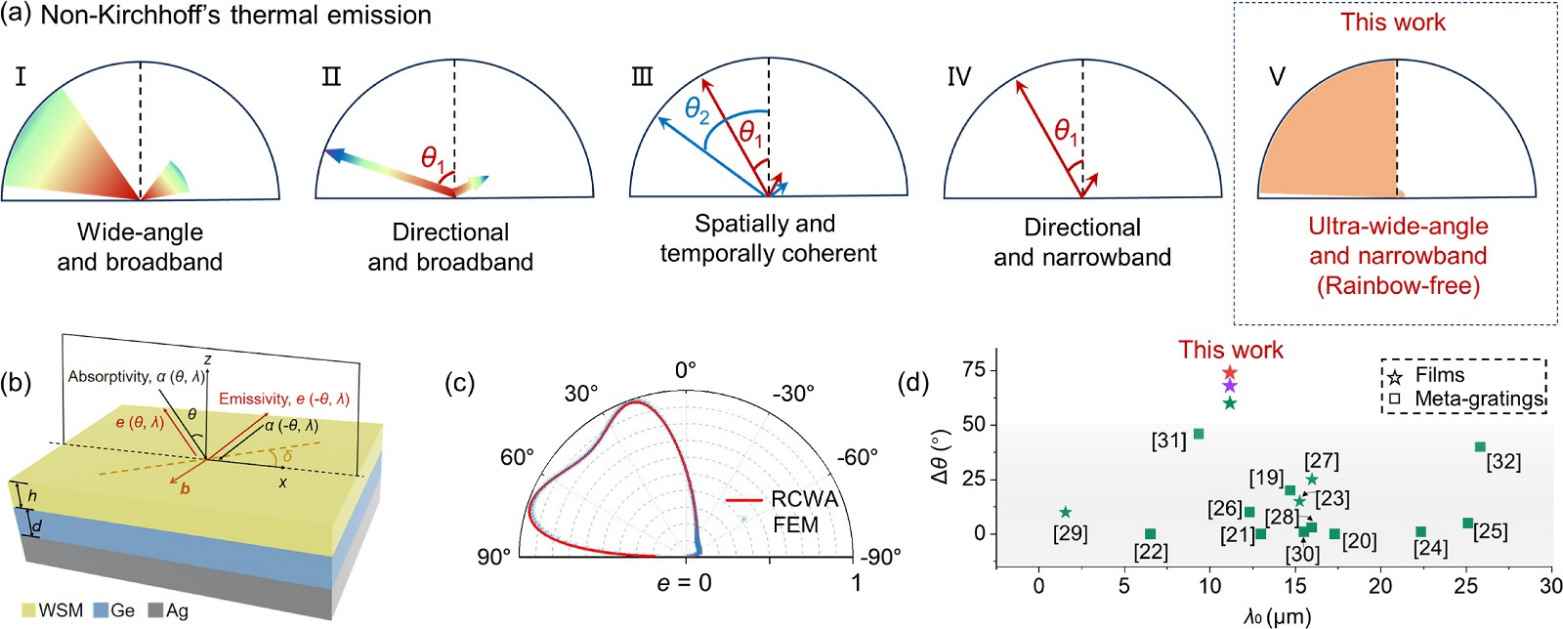
Fundamental characteristic of non‐Kirchhoff's thermal emission. (a) Conceptual illustration of nonreciprocal thermal emission. (b) Schematic of the proposed WSM/Ge/Ag structure with the polar angle *θ* and azimuthal angle *δ*. The thicknesses of WSM and Ge layers are denoted as *h* and *d*, respectively. (c) Angular emission profile at *λ* = 11.17 μm displayed in polar coordinates. (d) Performance comparison with state‐of‐the‐art narrowband nonreciprocal emitters [19, 20, 21, 22, 23, 24, 25, 26, 27, 28, 29, 30, 31, 32], where stars denote thin‐film designs and squares represent metasurfaces/grating‐based approaches, with colors reflecting different nonreciprocity thresholds (*η* > 0.8: green; *η* > 0.7: purple; *η* > 0.6: red).

The Fabry–Pérot (FP) resonator, consisting of a dielectric layer between two metal plates [38], offers a simpler alternative to complex nanostructures for achieving high absorptivity through resonance. However, conventional FP resonators exhibit a blue shift in resonance wavelength with increasing incident angle [38], which is a typical manifestation of the rainbow effect [34], creating a fundamental trade‐off between spectral purity and angular coverage [39]. Recent solutions employing high‐index or hyperbolic dielectrics [40], multilayer designs, or Tamm plasmons [41] have demonstrated angle‐invariant spectral selectivity, though they often come at the cost of increased structural complexity and thickness exceeding the operational wavelength.

Therefore, to simultaneously achieve narrowband, wide‐angle nonreciprocal emission, and compact device architecture, we propose a pattern‐free, rainbow‐free, and ultrawide angular nonreciprocal thermal emitter based on a minimalist WSM/Ge/Ag Fabry–Pérot resonator. The emitter maintains high emissivity (*e* > 0.8) over 7°–82° (i.e., Δ*θ* = 75°) and minimal spectral shift (Δ*λ/λ*_0_ < 0.54%) over 0°–89°, enabled by the WSM's unique phase shift compensation. Strong emission suppression at negative angles (0° to −89°) yields exceptional nonreciprocity. Active tuning is achieved through either Ge thickness or WSM chemical potential variation, while maintaining temperature‐insensitive operation, which is critical for practical applications. This work establishes an efficiency paradigm for angle‐robust nonreciprocal thermal control with potential applications in energy harvesting.

## 2 Results and Discussion

The proposed emitter consists of a Fabry–Pérot resonator with three distinct functional layers (Figure 1b): a WSM top layer, a germanium (Ge) dielectric spacer (*n* = 4), and a silver (Ag) back reflector. The WSM serves dual critical functions, enabling a strong nonreciprocal response through its intrinsic magneto‐optical properties and providing essential phase shift compensation. The Ag layer effectively suppresses transmission due to its near‐perfect conductivity, whereas the Ge layer forms an ideal low‐loss resonant cavity. A nonzero imaginary part of the dielectric function gives rise to absorption loss. As the medium is reciprocal, the difference in absorption of the proposed structure incorporating a lossy cavity between symmetric incidence angles at the same wavelength (i.e., η(λ,θ,δ)=α(λ,−θ,δ)−α(λ,θ,δ)) is reduced, which weakens nonreciprocity. Figure 1c shows the angular emission profile of our structure at the target wavelength in polar coordinates, with the results verified by both numerical methods. The emitter maintains high emissivity (*e* > 0.8) from 7° to 82° while strongly suppressing emission at negative angles. This demonstrates excellent nonreciprocal performance across a wide angular range within the narrow emission band. A comparative analysis with state‐of‐the‐art narrowband nonreciprocal emitters (Figure 1d) demonstrates our design's superior angular performance. $\lambda_{0}$ specifies the operating wavelength of each emitter, whereas Δθ denotes the angular range where the absolute difference between absorptivity and emissivity η exceeds predetermined thresholds: 0.8 (green), 0.7 (purple), and 0.6 (red). Although these systems achieve notable nonreciprocal performance, our design demonstrates superior angular robustness, maintaining |*α*‐*e*| > 0.6 across a remarkable 75° range, demonstrating an overwhelming advantage in nonreciprocity within an ultrawide‐angle range.

To obtain nonreciprocal behavior, we consider a transverse magnetic (TM) wave, where the electric field vector lies in the *x‐z* plane with polar angle *θ*. The azimuthal angle is defined as *δ*. The permittivity tensor of WSM can be expressed as follows [17, 42, 43]:

(1) $$\varepsilon =\left[\begin{array}{ccc} \varepsilon_{d} & 0 & i\varepsilon_{a}\\ 0 & \varepsilon_{d} & 0\\ -i\varepsilon_{a} & 0 & \varepsilon_{d} \end{array}\right],$$

where

(2) $$\varepsilon_{a}=\frac{be^{2}}{2\pi^{2}\hbar \omega \varepsilon_{0}},$$

(3) $$\varepsilon_{d}=\varepsilon_{b}+i\frac{\sigma }{\Omega_{0}},$$

2*b* is an axial vector along the *z* direction in momentum space, meaning that the separation between the two Weyl nodes is 2*b*. $\varepsilon_{0}$ is the permittivity of vacuum, $\varepsilon_{b}$ is the background permittivity, $\Omega_{0}=\hbar \omega /E_{F}$ is the normalized real frequency, and σ is the conductivity:

(4) $$\sigma =\frac{r_{s}g}{6}\Omega \;G\left(\frac{\Omega }{2}\right)+i\frac{r_{s}g}{6\pi }\left\{\frac{4}{\Omega }\left[1+\frac{\pi^{2}}{3}{\left(\frac{k_{B}T}{E_{F}}\right)}^{2}\right]+8\Omega \int_{0}^{\xi_{c}}\frac{G\left(\xi \right)-G\left(\frac{\Omega }{2}\right)}{\Omega^{2}-4\xi^{2}}\xi d\xi \right\}.$$

$\Omega =\hbar \left(\omega +i\tau^{-1}\right)/E_{F}$ is the normalized complex frequency, $E_{F}$ is the chemical potential, G(E)=n(−E)−n(E), $n\left(E\right)=1/\left[\exp\left(E‐E_{F}\right)/k_{B}T+1\right]$ is the Fermi distribution, $r_{s}=e^{2}/\left(4\pi \varepsilon_{0}\hbar v_{F}\right)$ is the Fermi velocity parameter, $v_{F}$ is the Fermi velocity, and g is the number of Weyl points [19].

According to Reference [19], $b=2\times 10^{9}\;m^{-1}$, $\varepsilon_{b}=6.2$, τ=1000fs, g=2, $\xi_{c}=3$, $v_{F}=0.83\times 10^{5}\;m\;s^{-1}$, *T* = 300 K [19]. Because the Fermi distribution can be tuned by electric gating, we set $E_{F}=0.20\;\text{eV}$ in this paper [17]. As Equation (1) reveals a nonzero off‐diagonal permittivity tensor with nonzero b, the scattering matrix becomes asymmetric, making Kirchhoff's law invalid. The permittivity of Ag is described by the Drude model: $\varepsilon =\varepsilon_{\infty }-{\omega_{p}}^{2}/\omega \left(\omega +i\Gamma \right)$, where $\varepsilon_{\infty }$, Γ, and $\omega_{p}$ are the high‐frequency permittivity, scattering rate, and plasma frequency, respectively. The relevant parameters are obtained from Reference [44].

As the thickness of the underlying Ag exceeds the skin depth, optical transmission approaches zero. Consequently, the emissivity e(λ,θ,δ) and absorptivity α(λ,θ,δ) can be calculated from the reflectivity for a given wavelength λ, polar angle θ, and azimuthal angle *δ*:

(5) $$e\left(\lambda ,\theta ,\delta \right)=1-R_{pp}\left(-\theta ,\lambda ,\delta \right)-R_{sp}\left(-\theta ,\lambda ,\delta \right),$$

(6) $$\alpha \left(\lambda ,\theta ,\delta \right)=1-R_{pp}\left(\theta ,\lambda ,\delta \right)-R_{ps}\left(\theta ,\lambda ,\delta \right),$$

where p and s denote *p*‐ and *s*‐polarized incident waves, respectively. The reflectance R is subscripted accordingly, where the first and second letters indicate the polarization states of the incident and reflected light, respectively (e.g., $R_{pp}$, $R_{ps}$, $R_{ss}$, $R_{sp}$).

The emissivity in this work is calculated by rigorous coupled wave analysis (RCWA) and is verified by the finite element method (FEM) implemented in COMSOL Multiphysics. Besides, the difference η between e(λ,θ) and α(λ,θ), which is used to describe the strength of nonreciprocity, can be obtained as follows:

(7) η(λ,θ)=e(λ,θ)−α(λ,θ).

To achieve optimal emissivity in the mid‐infrared regime, the thickness of the WSM layer (*h*) is set to 0.9 μm, and the thickness of Ge (*d*) is 1 μm.

To comprehensively characterize the angular and emission properties, we present the emissivity mapping in Figure 2a. The pronounced spectral feature (red band) reveals two key attributes: (i) a narrow resonance linewidth. The spectral emission peak maintains remarkable angular stability, exhibiting the rainbow‐free property with minimal wavelength shift (Δ*λ*/*λ*_0_ = 0.54%) across 0°–89°. The near‐horizontal orientation of this spectral feature at *λ*_0_ = 11.17 ± 0.06 μm demonstrates remarkable angular invariance of the resonant wavelength in nonreciprocal thermal emission systems. And (ii) significant angular asymmetry can be observed with respect to the zero angle, violating Kirchhoff's law. Specifically, at λ=10.80μm, emissivity at *θ* = 54° approaches 0, whereas emissivity at *θ* = − 54° becomes 0.9 (i.e., *α*(*θ* = 54°). Figure 2b presents the color map of η(λ,θ). The spectral emissivity and absorptivity exhibit pronounced nonreciprocal behavior across 10.60–11.17 μm, as evidenced by their significant angular divergence. Most strikingly, the spectral feature centered at 11.17 μm maintains η > 0.6 throughout an exceptionally wide angular range (7°–82°, i.e., Δ*θ* = 75°), directly demonstrating the emitter's strong nonreciprocal response that is insensitive to the polar angle, outperforming prior narrowband nonreciprocal designs. As derived in Equations (1) and (2), this fundamental nonreciprocal contrast originates from the nonzero off‐diagonal components of the dielectric tensor, which break the symmetry conditions required by Kirchhoff's law.

**FIGURE 2:**
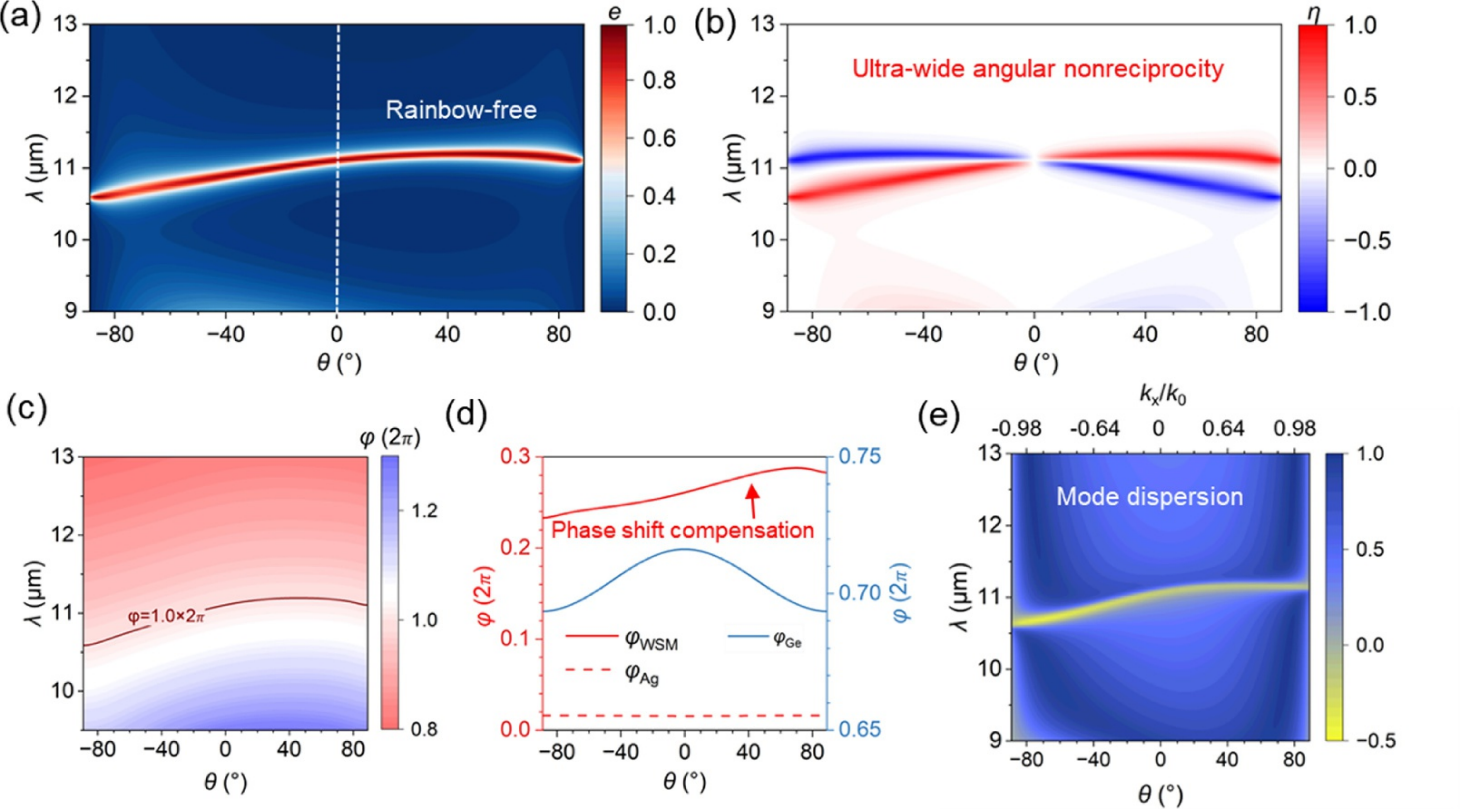
Thermal emission characteristics of the WSM/Ge/Ag structure. (a) Emissivity as a function of polar angle and wavelength. (b) Difference between emissivity and absorptivity as a function of polar angle and wavelength. (c) Total reflection phase shifts across the proposed structure. (d) Layer‐resolved contributions to the reflection phase shift, identifying the WSM layer's dominant role in phase shift compensation. (e) Mode dispersion for FP resonance, where *k*_x_ denotes the *x*‐axis component of the wavevector *k*_0._

The mechanisms behind narrowband and angular robustness can be explained based on the FP resonance condition. The FP resonance occurs when the wavelength and angle satisfy the following condition [45]:

(8) $$\varphi \left(\lambda ,\theta \right)=\varphi_{\text{WSM}}+\varphi_{\text{Ge}}+\varphi_{\text{Ag}}=2m\pi \;\left(m=0,1,2,3\text{…}\text{…}\right)$$

where φ(λ,θ) is the total reflection phase shift of the proposed structure, $\varphi_{\text{WSM}}$ is the reflection phase shift from Ge to WSM, $\varphi_{\text{Ag}}$ is the reflection phase shift from Ge to Ag, and $\varphi_{\text{Ge}}$ is the round‐trip propagation phase shift in the Ge cavity, which is given by the following equation:

(9) $$\varphi_{\text{Ge}}=2nd\;k_{0}\;\sin\;\theta ,$$

where $k_{0}$ is the wavevector in free space.

Phase analysis of the structure reveals critical insights into its angular robustness (Figure 2c,d). The total phase shift (red curve, Figure 2c), satisfying Equation (8)'s resonance condition, exhibits two key features: (i) single‐wavelength resonance excitation across all angles and (ii) remarkable insensitivity to the polar angle (0°–80°), directly explaining the angularly stable emission in Figure 2a. A layer‐resolved decomposition (Figure 2d) shows the Ag layer's minimal phase contribution ($\varphi_{\text{Ag}}$, red dashed line) and Ge's reciprocal phase reduction with increasing angle ($\varphi_{\text{Ge}}$, blue solid line). Both of them are reciprocal, and their accumulation decreases from 0° to 80°. The emergence of WSM breaks this pattern. This unique compensation mechanism maintains an essentially constant total phase shift, that is, φ=2π from 0° to 80°, as demonstrated by the red line in Figure 2c, thereby enabling the structure's remarkable wide‐angle nonreciprocal emission performance. In other words, at *λ* = 11.17 μm, the Fabry–Pérot resonance condition is satisfied across nearly all positive polar angles, enabling high emissivity. In contrast, negative angles exhibit fundamentally distinct behavior: $\varphi_{\text{Ag}}$, $\varphi_{\text{Ge}}$ and $\varphi_{\text{WSM}}$ all decrease monotonically with increasing angle (Figure 2d), preventing resonant enhancement over a broad angular range. This is because according to Equation (2), the chiral shift *b* ≠ 0 leads to a nonsymmetric dielectric tensor (*ε* ≠ *ε* ^T^, where *ε* ^T^ is the transpose of *ε*) for the TM wave. The inherent asymmetry of this tensor results in a nonreciprocal optical response, manifested as a change in the reflection coefficient when the sign of the polar angle is reversed at a fixed frequency. As $\varphi_{\text{WSM}}$ = arg(*r*_*pp*_), the phase shift of WSM $\varphi_{\text{WSM}}$ incredibly increases as θ increases from 0° to 80° (Figure 2d), which exhibits a counterintuitive angular‐dependent phase response that fundamentally differs from conventional materials. Hence, this broken symmetry directly demonstrates how WSM's nonreciprocity decouples the emission wavelength from angular dependence. Moreover, the dispersion relation of the FP resonance mode is illustrated in Figure 2e, which is calculated from the imaginary part of the reflection coefficient *r*_*pp*_. The positions where FP resonance occurs correspond precisely to those where *r*_*pp*_ exhibits extreme values, which exactly contribute to the bright spots in Figure 2e. Note that these dispersion curves of the resonance modes overlap with the contour plot in Figure 2a, demonstrating the important role of FP resonance modes in enhancing the nonreciprocal thermal emission.

Figure 3a demonstrates the rainbow‐free, wide‐angle nonreciprocal emission characteristics through spectral analysis. The emission spectrum exhibits a single resonant peak at λ=11.17μm for the polar angles of 15°, 45°, and 75°, with a Q factor of 90. While maintaining near‐unity emissivity (*e* > 0.98) at positive angles due to Fabry–Pérot resonance, the structure shows strongly suppressed emission (*e* < 0.25) at corresponding negative angles. This angular asymmetry leads to a high absorption rate contrast (> 98%) at *λ* = 11.17 μm between complementary angles, as predicted by Equation (7). Further insight comes from electromagnetic power dissipation density analysis [18, 22] (Figure 3b), which reveals markedly distinct energy dissipation patterns between symmetric polar angles, directly explaining the observed nonreciprocal behavior. As shown in Figure 3bI–III, energy dissipation predominantly occurs at the bottom surface of the WSM layer and the top surface of the Ag layer, where resonant cavity effects enhance light–matter interaction through multiple reflections between the two metallic layers. This extended dwell time leads to complete energy absorption in the lossy metallic components. In contrast, Figure 3bIV–VI shows minimal energy loss as light at λ=11.17μm and *θ* < 0° fails to satisfy the resonance condition (Figure 2d), demonstrating the structure's angular‐selective absorption/emission.

**FIGURE 3:**
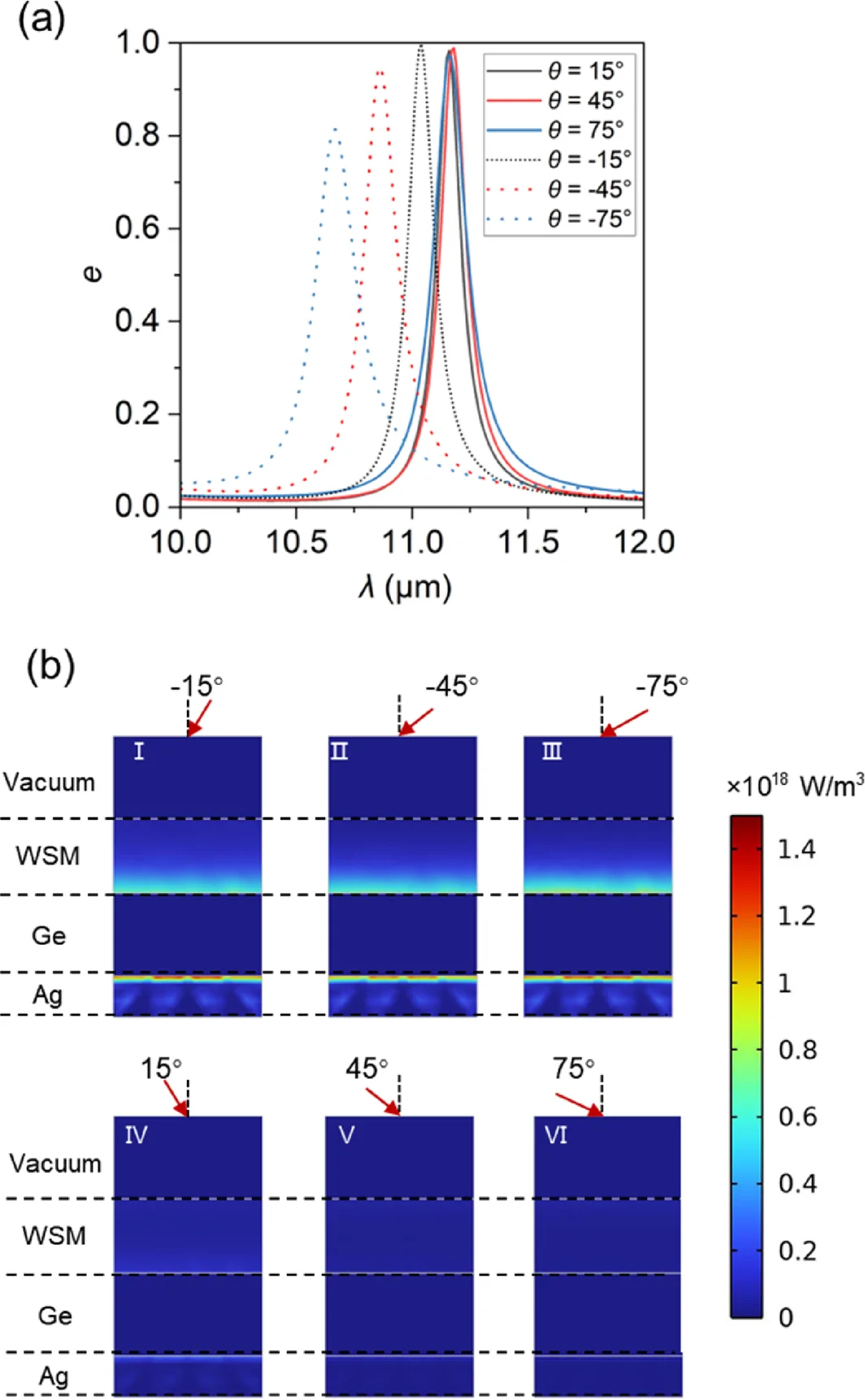
(a) Emissivity as a function of wavelength for polar angles of ±15°, ±45°, and ±75°. (b) Distribution of electromagnetic power dissipation density of the proposed structure at 11.17μm for polar angles of ±15°, ±45°, and ±75°.

We investigate the spectral and angular emission characteristics as functions of the WSM's chemical potential (*E*_F_) and Ge spacer thickness. The emission peak undergoes a continuous blueshift from 11.17 μm to 10.51 μm with increasing *E*_F_ (0.20–0.23 eV) not by varying the temperature but by adjusting the doping concentration, as shown in Figure 4a–d. The rate of the wavelength change (μm eV^−1^) is up to 23 (calculated as 0.66 μm/0.03 eV). This tunability originates from *E*_F_‐dependent phase modulation in the WSM layer that modifies the Fabry–Pérot resonance condition, in agreement with Equation (8). We have also noted that some hybrid graphene Weyl semimetal structures can offer spectral control at specific incident angles with a rate of wavelength change of 4 [46, 47]. Furthermore, our structure can be designed according to the target wavelength while maintaining its nonreciprocal monochromatic and wide‐angle characteristics, as shown in Figure 4a–d. As the Ge thickness increases from 0.9 μm to 1.2 μm, the emission peak redshifts by 1.95 μm to 12.51 μm (Figure 4e–h). This behavior follows directly from the FP resonance condition in Equation (9): Increasing the cavity thickness *d* requires a proportional increase in wavelength *λ* to maintain constructive interference. Notably, the spectral line shape remains invariant during tuning, preserving both wide‐angle operation and rainbow‐free performance across all tested parameter variations. This insensitivity in maintaining nonreciprocal characteristics under spectral tuning demonstrates the structure's potential for adaptable thermal emission control.

**FIGURE 4:**
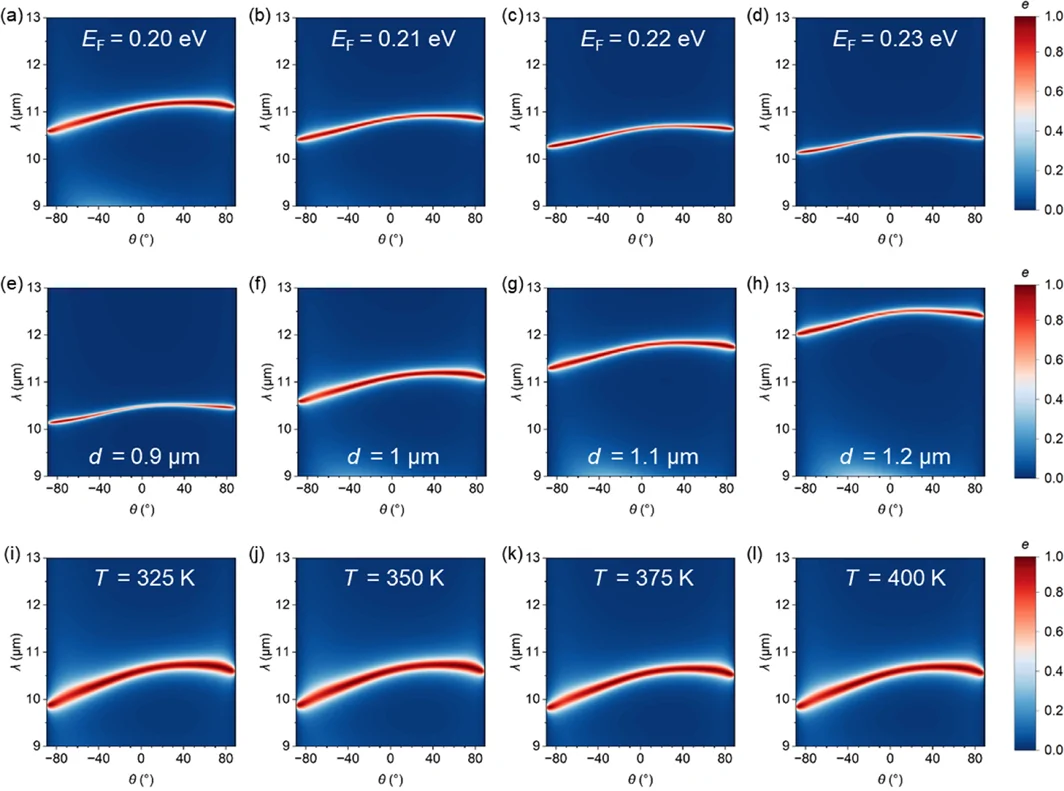
Angular robustness in controllable nonreciprocal thermal emission. (a–d) Emissivity as a function of polar angle and wavelength at chemical potentials of 0.20, 0.21, 0.22, and 0.23 eV. (e–h) Geometrical tuning via Ge spacer thickness variation of 0.9, 1.0, 1.1, and 1.2 μm. (i–l) Temperature‐dependent performance, where *T* = 325, 350, 375, and 400 K, respectively.

Temperature changes alter the chemical potential, as expressed in the following equation [19]:

(10) $$E_{F}\left(T\right)=\frac{2^{\frac{1}{3}}{\left[9E_{F}{\left(0\right)}^{3}+\sqrt{81E_{F}{\left(0\right)}^{6}+12\pi^{6}k_{B}^{6}T^{6}}\right]}^{\frac{2}{3}}-2\pi^{2}3^{\frac{1}{3}}k_{B}^{2}T^{2}}{6^{\frac{2}{3}}{\left[9E_{F}{\left(0\right)}^{3}+\sqrt{81E_{F}{\left(0\right)}^{6}+12\pi^{6}k_{B}^{6}T^{6}}\right]}^{\frac{1}{3}}},$$

where *E*_F_(0) = 0.2104 eV, which yields *E*_F_(300K) = 0.2000 eV in this work. Temperature affects *E*_F_ but has little effect on conductivity *σ* across the 325–400‐K range (analogous to graphene's thermal behavior [48, 49, 50, 51]), resulting in negligible variations in both spectral linewidth and peak wavelength position (< 0.72%), as evidenced in Figure 4i–l. This robust temperature independence guarantees stable nonreciprocal operation under realistic thermal conditions.

Finally, we investigate the emissivity at 11.17 μm in the plane with the azimuthal angle δ ranging from 0° to 360°. The structure maintains high emissivity (*e* > 0.8) across a wide angular range for *δ* ∈ [−330°, 30°], while showing markedly reduced emission at complementary azimuthal orientations (δ+180° in Figure 5a). This pronounced directional anisotropy originates from the angle‐dependent coupling between the Weyl node separation vector ***b*** and the electric field polarization.

**FIGURE 5:**
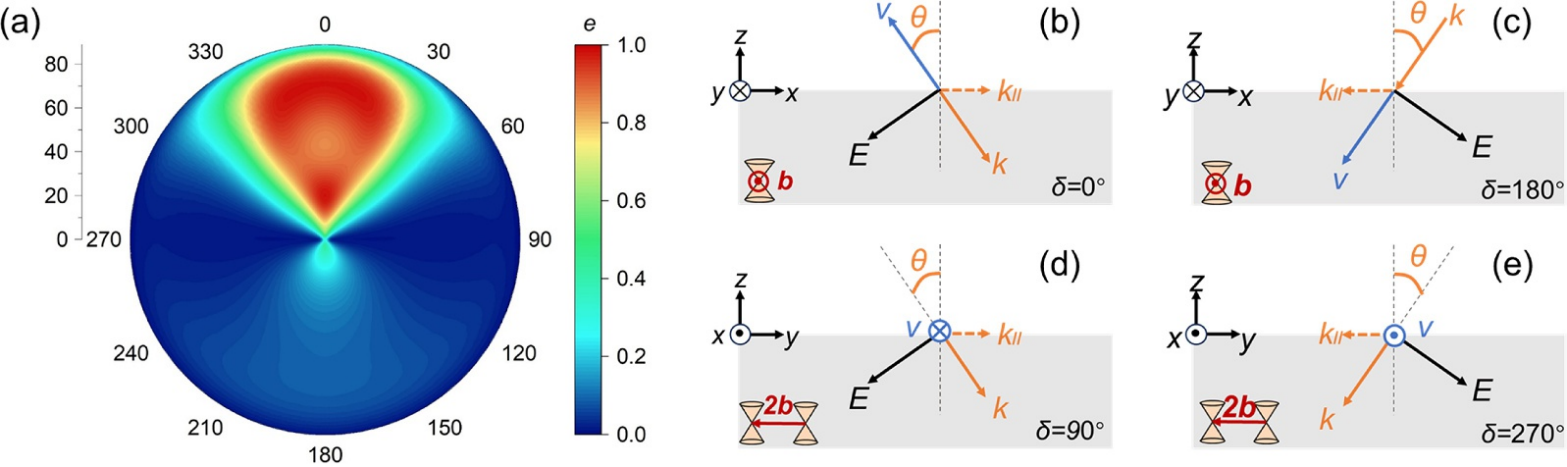
The influence of the azimuthal angle *δ*. (a) Emissivity as a function of polar angle and azimuthal angle at 11.17 μm. (b) *v* slows down each Weyl fermion moving along *k*_//_ for *δ* = 0°, where *k*_//_ is the in‐plane component of the wavevector. (c) *v* speeds up each Weyl fermion moving along *k*_//_ for *δ* = 180°. (c) *v* is perpendicular to *k*_//_ for *δ* = 90°. (d) *v* is perpendicular to *k*_//_ for *δ* = 270°.

The constitutive relation in the frequency domain for the Weyl semimetal is given as follows [52]:

(11) $$D=\varepsilon_{d\;}E+\frac{ie^{2}}{4\pi^{2}\hbar \omega }\left(-2b_{0}B+2b\times E\right),$$

where ***D*** denotes the electric displacement field, ***E*** is the electric field, ***B*** is the magnetic field, and $b_{0}=0$ in this work.

***D*** is composed of the Dirac term and the axion term. The latter implies that under an electric field ***E***, electrons acquire an anomalous Hall velocity ***v*** perpendicular to ***E*** $\times \Omega^{\prime }\;($the Berry curvature $\Omega^{\prime }$ is parallel to 2 ***b***), which constitutes the intrinsic mechanism of the anomalous Hall effect. Treating the charge carriers as semiclassical wave packets of Weyl fermions [53], ***v*** exerts different effects on Weyl fermions moving along the *k*_//_ direction depending on the azimuthal angle *δ.* ***v*** retards their motion when *δ* = 0° (Figure 5b), while accelerating them when *δ* = 180° (Figure 5c), leading to the nonreciprocity of Weyl semimetals. However, in both cases of *δ* = 90° (Figure 5d) and 270° (Figure 5e), the anomalous velocity is perpendicular to the *x*‐direction and therefore has no component along the direction of motion, suppressing nonreciprocity, as confirmed by Figure 5a.

The feasibility of growing this structure has been experimentally established. This advancement builds upon the discovery of Weyl semimetals (WSMs) such as TaAs [54], TaP [55], and NbP [56] since 2015, with a notable example being the successful growth of tantalum arsenide (TaAs) thin films using molecular beam epitaxy and deposition techniques [57, 58, 59]. Subsequently, the Ge and Ag layers [50, 51, 52, 53, 54, 55, 56, 57, 58, 59, 60, 61, 62] can be deposited onto the doped WSMs using standard atomic layer deposition or E‐beam evaporation methods [55, 56, 57]. Different types of dopants can be injected into WSMs to increase the number of free charge carriers, enabling precise chemical potential control [63, 64]. The emissivity and absorptivity can be measured by a home‐built setup [10, 21, 65]. Emission and absorption from the structure are sent to a Fourier transform infrared spectrometer (FTIR) for measurement after the structure itself is heated and rotated to preset conditions.

## 3 Conclusion

In summary, we propose a pattern‐free emitter comprising Weyl semimetal/germanium heterostructures on a silver substrate that achieves rainbow‐free nonreciprocal thermal emission with exceptional angular stability. As a result, phase analysis reveals the operational mechanism of our emitter, highlighting the Weyl semimetal's crucial role in phase shift compensation. The Weyl semimetal enables the emitter's exceptional performance through two synergistic mechanisms: breaking Lorentz reciprocity via its large gyrotropic optical responses while simultaneously providing dynamic phase shift compensation. This dual functionality maintains strong nonreciprocity (*η* > 0.6 or 0.8) across an unprecedented angular range (Δ*θ* = 75° or 60°) at the design wavelength of 11.17 μm with monochromaticity of Δ*λ*/*λ*_0_ < 0.54%, delivering superior angular robustness using a simpler structure compared to previous narrowband nonreciprocal emitters. Spectral tuning is achieved through either chemical potential modulation of the Weyl semimetal or precise adjustment of the cavity thickness, with both methods preserving the emitter's spectral selectivity and wide‐angle characteristics, thereby establishing a generalizable design framework for achieving target performance at other wavelengths. Remarkably, the nonreciprocal performance shows negligible degradation under temperature fluctuations (Δ*T* = 100 K) or azimuthal variations (Δ*δ* = 60°). This work establishes a simplified, cost‐effective platform for nonreciprocal thermal emission that maintains high performance while eliminating complex nanostructuring requirements. The approach based on Fabry–Pérot (FP) resonance remains valuable, as it offers both a clear physical explanation and a practical guide for device development. The underlying phase equations, which are both general and concise, provide a feasible basis for designing structures across different wavelength regimes. Furthermore, their simplicity should establish a straightforward and effective foundation for integrating machine learning into the design process in the future.

## Author Contributions

**Kaixia Xu:** conceptualization, formal analysis, investigation, methodology, validation, software, writing – review and editing, writing – original draft. **Kezhang Shi:** conceptualization, data curation, funding acquisition, formal analysis, investigation, methodology, resources, writing – review and editing, supervision, software, visualization, validation. **Meiya Rong:** formal analysis, software, data curation. **Run Hu:** conceptualization, formal analysis, project administration, methodology, resources, validation, supervision, writing – review and editing. **Chengping Yin:** conceptualization, formal analysis, project administration, resources, methodology, writing – review and editing, validation, supervision.

## Conflicts of Interest

The authors declare no conflicts of interest.

## Data Availability

The data that support the findings of this study are available from the corresponding author upon reasonable request.
